# The 10 Commandments for Transapical TAVI

**DOI:** 10.1177/15569845231177052

**Published:** 2023-06-13

**Authors:** Gry Dahle, Thomas Walther

**Affiliations:** 1 Department of Cardiothoracic Surgery, Oslo University Hospital, Norway; 2 Klinik für -Herz- und Gefässchirurgie, Universitätsklinikum Frankfurt, Germany

## Introduction

In 1992, Henning Ruud Andersen carried out the first transcatheter implantation of an artificial aortic valve in pigs.^1^ However, the idea of implanting balloon-expandable stented valves in humans was met with skepticism until the cardiologists Cribier and Eltchaninoff performed the first-in-human transcatheter aortic valve implantation (TAVI) in 2002.^2^ This was done through transfemoral (TF) transvenous access with a transseptal approach to the left ventricle and then antegradely through the aortic valve. In those early days of TAVI therapy, the size of the delivery sheath was quite large; thus, alternative access routes were investigated, leading to the first reports of transapical (TA) TAVI. This method was further elaborated on and for some years was the access route of choice at several centers.^3^ In the years 2012 to 2013, in parallel to refinements of the delivery sheaths, especially due to the lower profiles, led to an almost equal number of TF and TA procedures being performed. Later on, the TF procedure under local anesthesia was introduced and thus became more advantageous. Today, the vast majority of TAVI procedures are performed through a TF approach. However, there are clinical conditions in approximately 5% of patients who suffer severe peripheral vascular disease, and thus TA access should be chosen. Advantages of TA access are the antegrade approach, the ease of valve crossing, and the excellent control during valve deployment.^4^ The procedure was also found to be suitable for valve-in-ring mitral^5^ and at present is the main access route for transcatheter mitral valve implantation (TMVI).^6^ When evaluating individual patient profiles in the Heart Team, we believe there are 10 commandments that should be recognized in order to perform a safe TAVI procedure.

## 1. Choose the Right Access for Each Individual Patient

**Fig. 1.**
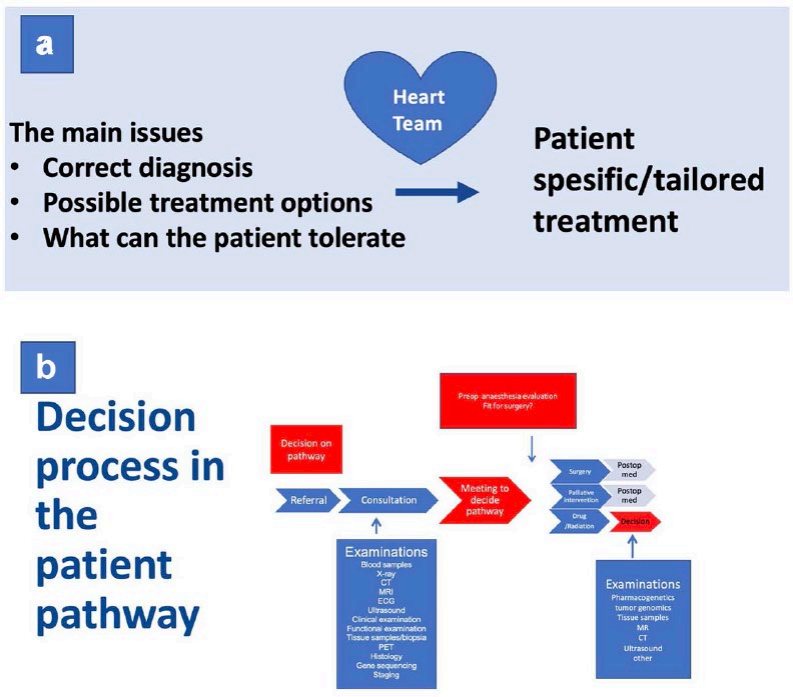
(a) The process of patient-specific treatment. (b) The decision process form referral to treatment.

Prior to TAVI, it is important to perform relevant anatomical screening and risk assessment. Echocardiography and computed tomography (CT) scan (with reconstructions for access route, annulus size, and implant angles) as well as exclusion of coronary artery disease, plus additional exams such as pulmonary function test, frailty score, kidney function, and so forth should be assessed. All of these parameters have to be discussed in the Heart Team to decide on indication and the best access route for the specific patient (Fig. 1).

TAVI prostheses include expandable valves such as CoreValve/Evolut™, Acurate Symetis™, or Navitor™ or a ballon-expandable SAPIEN device. The SAPIEN 3 system at present is the only TAVI prosthesis that is available for an antegrade TA delivery.

## 2. Good Procedural Planning Is Crucial for a Good Result, as With TF TAVI

In the early years of TAVI, sizing was done by echocardiography, and when in doubt, sizing was corrected after performing balloon valvuloplasty. Implantation angles were set periprocedurally; hence, procedural and radiation time were longer and contrast volume higher.

**Fig. 2.**
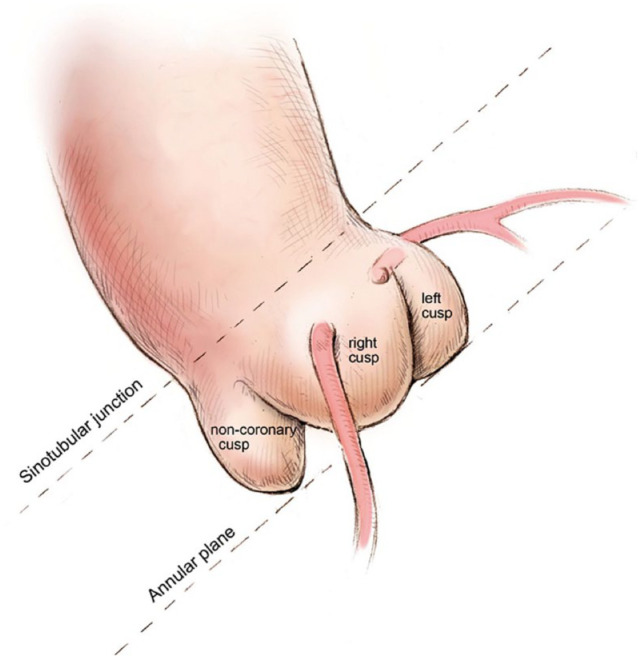
The bases of all three aortic cusps residing on the same plane is crucial to a successful implantation. Used with permission of AME Publishing Company, from “Illustrated Techniques for Transapical Aortic Valve Implantation,” Cheung and Lichtenstein, 1(2), 2012; permission conveyed through Copyright Clearance Center, Inc.^7^

The procedure can (and should) nowadays be planned regarding valvular sizing and implant angles by analyzing the reconstructed CT images. CT angiography of the heart, aorta, and iliac arteries down to the groin is the standard imaging method for preinterventional evaluation and planning of the TAVI procedure. The CT examination provides detailed information about the size and geometry of the aortic annulus. The annulus is a virtual ring formed from the most basal attachment points of all 3 aortic valve cusps (Fig. 2).^7^ Measurement of the aortic annulus is essential for selecting the appropriate prosthesis.^8^ In addition, the implant angles are calculated. This will reduce the procedural time, radiation exposure to operators and patients, and reduce use of contrast.

**Fig. 3.**
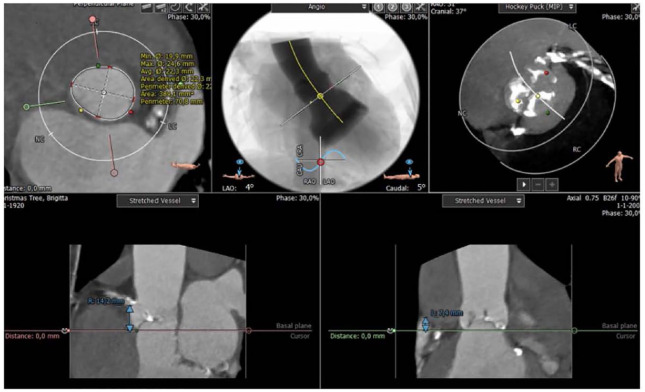
Computed tomography reconstructions for procedural planning with annulus sizing, implant angle, calcium distribution, and distance to coronaries.

The planning is performed in specific CT software such as OsiriX™ or 3Mensio™ (Fig. 3).^9,10^ In addition, the FeOps™ system could be used to calculate the potential tissue deformation by the valve.^11^ From this, patient-specific parameters can be analyzed, and individual planning can be done. Machine learning and artificial intelligence will further help to improve image quality and thus lead to an even safer procedure.^12^

## 3. TAVI Procedures Should Be Performed in a Hybrid Operation Room

A hybrid operation room is a surgical theater that is equipped with advanced medical imaging devices such as fixed high-resolution C-arms, in addition to echocardiography machines. A hybrid suite allows combined open surgical and interventional procedures. A hybrid suite facilitates conversion from closed interventions to open surgical procedures when required, as the room is fully equipped with cardiac surgical tools including a heart–lung machine.

**Fig. 4.**
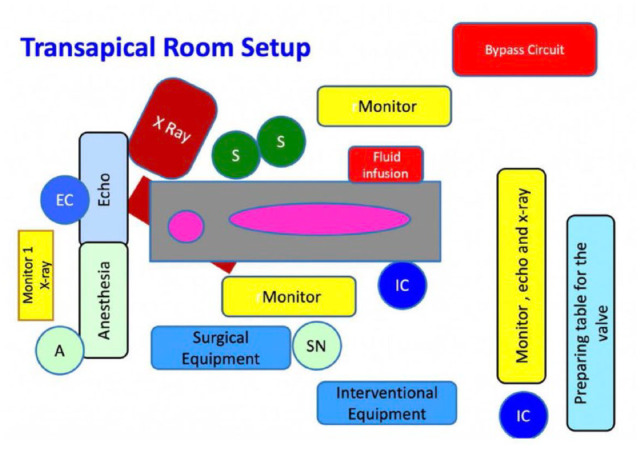
Hybrid room set up for transapical procedure. The surgeons on the left side of the patient and monitor, scrub nurse, radiographer, and interventional cardiologist at their opposite side.

The room set up for TA TAVI has to be modified from the TF set up to be functional (Fig. 4). The patient is under general anesthesia. Transesophageal echocardiography may be used for guidance during the procedure. The echocardiographer and the anesthetist are positioned at the head of the patient. The surgeons are on the left side of the patient, and the scrub nurse, interventional cardiologist, and surgical and interventional equipment are on the right side. There should also be a monitor on the right side. A bypass circuit for extracorporeal membrane oxygenation or a heart–lung machine should be in the room behind the surgeons. The preparing table for the valve may be placed as for the TF approach.

## 4. Exposure of the Apex and Precise Apical Suturing Are Critical for Successful TA TAVI

**Fig. 5.**
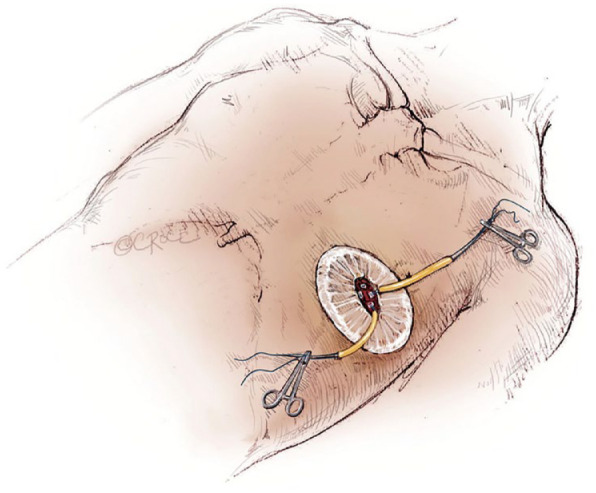
A 3 cm incision is made over the sixth intercostal space and a soft tissue retractor, Alexis Retractor (Applied Medical Corp., Rancho Santa Margarita, CA, USA) is inserted into the incision to retract the soft tissue without spreading the ribs. Used with permission of AME Publishing Company, from “Illustrated Techniques for Transapical Aortic Valve Implantation,” Cheung and Lichtenstein, 1(2), 2012; permission conveyed through Copyright Clearance Center, Inc.^7^

The exact position of the left ventricular apex is visualized in relation to the patient’s chest by transthoracic echocardiography. The CT scan may indicate the level of incision. An anterolateral left minithoracotomy is performed. A soft tissue retractor is inserted with or without an additional rib retractor (Finochietto) to give good exposure (Fig. 5).^3,7^ An even better exposure to the apex may be obtained with the soft tissue retractor inside the pericardium. From the CT scan, the implantation view is precalculated.

**Fig. 6.**
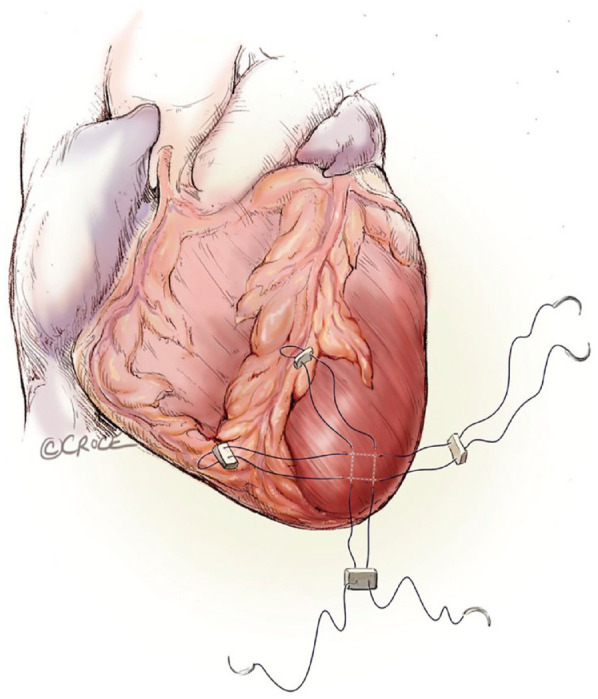
Two large pledgetted orthogonal mattress sutures using 3-0 MH polypropylene sutures (Ethicon, Somerville, NJ, USA) to obtain full thickness of the left ventricular wall and each of the two mattress sutures are snared and passed through tourniquets that can be tensioned at the time of sheath removal. Used with permission of AME Publishing Company, from “Illustrated Techniques for Transapical Aortic Valve Implantation,” Cheung and Lichtenstein, 1(2), 2012; permission conveyed through Copyright Clearance Center, Inc.^7^

The pericardium is opened, and stay sutures are placed. In a redo situation, the pericardium is left intact. Usually, 2 pairs of pledget U-sutures perpendicular to each other are sufficient, using Prolene 3-0 with a rather big needle (MH/SH; Fig. 6).^7^ The stitches are placed only partially into the myocardium rather than completely through the ventricular wall, to avoid bleeding or intramural hematoma. A double purse-string suture with somewhat smaller pledgets (3 × 7 mm) may also be useful in some cases.

**Fig. 7.**
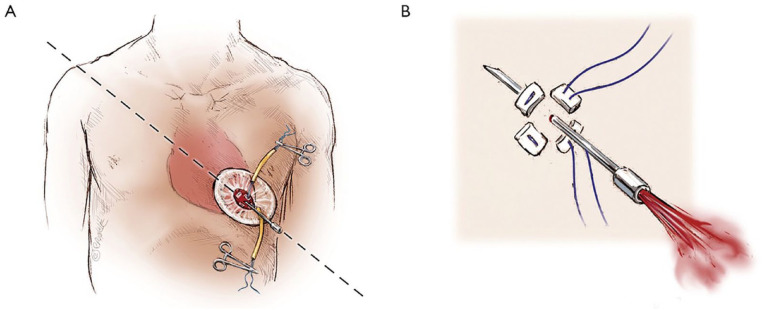
(A) A 14-gauge Seldinger needle is positioned in the center of the mattress sutures square, advanced to enter the chamber of the left ventricle towards the right shoulder, whereby crossing of the native aortic valve can easily be achieved. (B) Correct placement can be confirmed by the visualization of bright red blood spurting with each ventricular contraction. Used with permission of AME Publishing Company, from “Illustrated Techniques for Transapical Aortic Valve Implantation,” Cheung and Lichtenstein, 1(2), 2012; permission conveyed through Copyright Clearance Center, Inc.^7^

The apical puncture is made in the middle of the purse string, and it is important to have good backflow of oxygenated blood. It is possible to hit the septum or the right ventricle, and there will then be no backflow or backflow with deoxygenated blood (Fig. 7).^7^

## 5. TA Valve Delivery Is Antegrade and Fast: Be Aware of Correct Loading Position

**Fig. 8.**
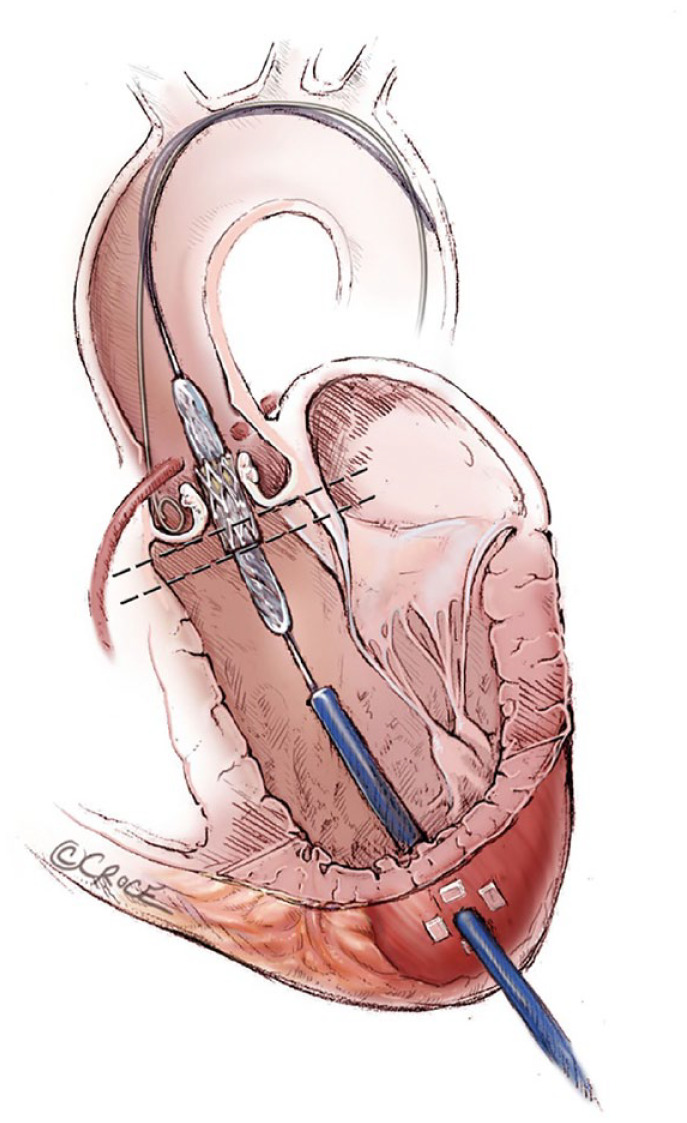
The SAPIEN prosthesis is ideally placed 1/3 below the base of aortic sinuses, the bottom of the valve stent ventricularly relative to the line of perpendicularity with the aid of repeat aortic root angiograms. Used with permission of AME Publishing Company, from “Illustrated Techniques for Transapical Aortic Valve Implantation,” Cheung and Lichtenstein, 1(2), 2012; permission conveyed through Copyright Clearance Center, Inc.^7^

It is important to load the valve in an antegrade position, opposite from TF, with the skirt proximal on the balloon. It is the operator’s responsibility to check this (Fig. 8).^7^ Pacing leads may be placed directly on the apex or via the jugular vein. An angiographic catheter can be placed from the radial artery or femoral artery; many patients with TA access are very arteriosclerotic. The apex puncture is usually not done on the true apex but more anteriorly on the naked myocardium in the triangle between the left anterior descending artery and the most distal diagonal branch. Puncture in an area covered by a larger amount of epicardial fat should be avoided. The guide wire is inserted antegradely through the aortic valve; it usually passes quite directly. If the correct C-arm position is predetermined, then the implantation is done very quickly with minimum use of contrast and radiation time. The exact positioning is easy and controlled, as the distance from the apex to the valve is 7 to 10 cm; hence, there is no delay in movement. Transesophageal echocardiography may be performed in addition to angiogram to evaluate the right position and eventually exclude any paravalvular leak. The sheath is removed, and the purse strings are tightened; at this moment, it is of great importance that the systolic pressure is not too high, ideally around 100 mm Hg. The pericardium, if opened, may be tied by the stay sutures. A chest drain is placed. The intercostal space should be closed by approximating the adjacent ribs with a suture. It is important to close the chest properly, especially if the patient is thin, to avoid subcutaneous emphysema. The safety of the TA procedure was shown in the PREVAIL TA study on 150 patients, of whom only 1 patient (0.7%) suffered an access-related complication.^13^

## 6. An Effective Closure Device Is Not Constructed

There have been many attempts to construct a safe and effective apical closure device. None of these have been implemented for a longer duration. Animal studies were done for self-expandable plugs and also some kinds of suture systems; very few were found safe enough to continue in humans.^14^ The APICA ASC™ titanium coil (Apica Cardiovascular Limited, Galway, Ireland) was applied in more than 100 humans; however, after the study, it was not further continued.^15^

## 7. Not All Valve Systems Have TA Delivery

Depending on the delivery system, some valves are suitable for TA TAVI and some are not. The CoreValve/Evolut™ (Medtronic, Dublin, Ireland) and Portico/Navitor™ (Abbott, Chicago, IL, USA) do not have TA delivery. The Acurate Symetis™ prosthesis (Boston Scientific, Marlborough, MA, USA) had been used with an apical delivery system; however, this route was not continued further. In fact, it is currently only the balloon-expandable platform such as the SAPIEN valve that has delivery systems for TA access. The Acurate Symetis™ and Trilogy (JenaValve, Irvine, CA, USA) are now available for TF delivery.

## 8. TA Provides Good Access for Mitral Valve-in-Ring and Valve-in-Valve and Native TMVI

The TA access is perfect for reaching the mitral valve for valve-in-ring, valve-in-valve, and implanting in the native mitral annulus. The same precautions as for other TMVI have to be taken to avoid left ventricular outflow tract (LVOT) obstruction, which means calculating neoLVOT with the valve implanted, in both diastole and systole. However, the delivery is very accurate and fast. So far, only 1 transcatheter mitral valve prosthesis is commercially available, the Tendyne™ (Abbott). The delivery sheath is 36Fr because of the large size of the valve, and the valve is secured by a tether and apical pad; hence, the delivery has to be TA. As such, the TA approach right now is the safest, easiest, and most logical direct approach to reach the mitral valve, especially for TMVI.

## 9. Be Prepared to Deal With Complications

During TAVI, anything can happen, anytime. Therefore, the procedure should be performed by an experienced team in a hybrid operation suite, with a heart–lung machine in the room and perfusionist available. Surgical equipment and a scrub nurse should be available. Adrenalin (0.1 mg/mL) should be in a syringe at the operation table, ready to use in case of emergency (unstable hemodynamics with hypotension). Blood products should be available.

The worst complication to TA TAVI is apical rupture. The most important thing in these situations is to unload the ventricle and lower blood pressure. Access sheaths in the femoral artery and vein may be useful for fast cannulation to go on pump. It can also be useful to expand the thoracotomy to access the unloaded ventricle for repair (on beating heart). Felt sutures may then be placed to repair the apex. In case cardiac arrest is required, the team may consider conversion to sternotomy to obtain larger exposure. Other complications may occur, but these are also the fact for TF procedures such as annulus rupture, valve embolization, coronary obstruction, and atrioventricular block.

## 10. Postoperative Follow-Up and Treatment Are Important and Should Be Done Similar to All Other Patients After Aortic Valve Procedures

The patients are treated for aortic stenosis. They have a thick and stiff ventricle and need to be well filled. It is also of importance to avoid atrial fibrillation (optimize electrolytes, hemoglobin). During the first postoperative hours, the blood pressure should be stable—not too high but also not too low. In patients with left ventricular hypertrophy, sufficient ventricular filling is required.

The chest drain should be removed on the first or second postoperative day to avoid pain for the patient. It is important that the patient is mobilized and receives physiotherapy with specific focus on respiratory support to avoid atelectasis and pneumonia. Regarding anticoagulation, the use of antiplatelet treatment is a matter of discussion. Some centers give single antiplatelet therapy. Some centers prefer Coumadin for the first 3 months, especially if the patient is in atrial fibrillation. Novel oral anticoagulants are not recommended. It may be a problem if the patients need drainage for pleural effusion or other interventions and there is little evidence for preventing valve thrombosis.

## Conclusions

TA access is a routine approach that has been used for antegrade TAVI implantation for many years. At present, it remains the most frequently used access modality for TMVI. Advantages are the ease of use and the direct access to the left ventricle, together with the aortic and mitral valves. Routine use of the TA approach leads to good results, which in some studies have been quite comparable to TF TAVI procedures. However, with the development of smaller delivery sheaths, the number of TA TAVI implants declined, and the number of TF implants consecutively increased to almost 95% of all procedures. TA access is a safe alternative for TAVI for selected patients, although it is the most fragile and morbid patients who are offered this access approach; hence, the morbidity will be higher than for TF. As such, the TA approach is a valuable tool and access modality to treat higher risk patients with valve disease by the heart team.
